# Phylogenetic study documents different speciation mechanisms within the *Russula globispora* lineage in boreal and arctic environments of the Northern Hemisphere

**DOI:** 10.1186/s43008-019-0003-9

**Published:** 2019-06-07

**Authors:** Miroslav Caboň, Guo-Jie Li, Malka Saba, Miroslav Kolařík, Soňa Jančovičová, Abdul Nasir Khalid, Pierre-Arthur Moreau, Hua-An Wen, Donald H. Pfister, Slavomír Adamčík

**Affiliations:** 1 Department of Cryptogams, Institute of Botany, Plant Science and Biodiversity Centre, Slovak Academy of Sciences, Dúbravská cesta 9, SK-845 23 Bratislava, Slovakia; 2 State Key Laboratory of Mycology, Institute of Microbiology, Chinese Academy of Sciences, No 3 1st Beichen West Road, Chaoyang District, Beijing, 100101 China; 3 Department of Plant Sciences, Quaid-i-Azam University, Islamabad, 45320 Pakistan; 4 Department of Botany, University of the Punjab, Quaid-e-Azam Campus, Lahore, 54590 Pakistan; 5 Laboratory of Fungal Genetics and Metabolism, Institute of Microbiology, Czech Academy of Sciences, Vídeňská 1083, CZ-142 20 Praha, Czech Republic; 6 Department of Botany, Faculty of Natural Sciences, Comenius University in Bratislava, Révová 39, SK-811 02 Bratislava, Slovakia; 7 Laboratoire IMPECS, Fac. Pharma. Lille, Université de Lille, F-59000 Lille, France; 8 Farlow Reference Library and Herbarium of Cryptogamic Botany, Harvard University, Cambridge, MA 02138 USA

**Keywords:** Ectomycorrhizal fungi, Biogeography, Climate, Disjunction, Evolutionary drivers, New taxa

## Abstract

The *Russula globispora* lineage is a morphologically and phylogenetically well-defined group of ectomycorrhizal fungi occurring in various climatic areas. In this study we performed a multi-locus phylogenetic study based on collections from boreal, alpine and arctic habitats of Europe and Western North America, subalpine collections from the southeast Himalayas and collections from subtropical coniferous forests of Pakistan. European and North American collections are nearly identical and probably represent a single species named *R. dryadicola* distributed from the Alps to the Rocky Mountains. Collections from the southeast Himalayas belong to two distinct species: *R. abbottabadensis* sp. nov. from subtropical monodominant forests of *Pinus roxburghii* and *R. tengii* sp. nov. from subalpine mixed forests of *Abies* and *Betula*. The results suggest that speciation in this group is driven by a climate disjunction and adaptation rather than a host switch and geographical distance.

**Electronic supplementary material:**

The online version of this article (10.1186/s43008-019-0003-9) contains supplementary material, which is available to authorized users.

## INTRODUCTION

*Russula* is a cosmopolitan genus of basidiomycetes comprising hundreds of species with a mainly agaric habit of the basidiomes (Looney et al. 2016). Members of the genus are important ectomycorrhizal (ECM) partners in forest ecosystems and are also used as commercially traded edible fungi (Looney et al. 2018). A study of global datasets of *Russula* shows only small overlaps in species diversity between geographically distant areas in the Northern Hemisphere (Looney et al. 2016). Current studies suggest that *Russula* species reported from multiple continents are mainly boreal-arctic taxa. Bazzicalupo et al. (2017) found that 17 of 72 *Russula* species from the Northwest of the USA (mostly from boreal-arctic areas) matched a sequence of European barcoded taxa. Accordingly, Geml et al. (2012) suggested that long distance dispersal and gene flow between boreal-arctic species of the *Russulaceae* (*Russula* and *Lactarius*) was common. A phylogenetic study of subsection *Xerampelinae* (Adamčík et al. 2016a) has demonstrated that *R. subrubens* and *R. clavipes* occur not only in distant areas of Europe, but that they also inhabit a wide range of ecosystems in very different climatic areas (from the temperate belt to boreal-arctic-alpine regions).

Little is known about the phylogeography of ECM fungi in Eurasia. For a long time, European *Russula* species were believed to be present in East Asia. Hongo (1960), for example, listed only four native Asian species among 40 Russulas reported from Japan, and 31 species reported from the country were originally described from Europe; the remaining species were from North America. Cao et al. (2013) demonstrated a 3–6% ITS nrDNA sequence divergence between Chinese collections and the most similar European species *R. virescens*, but despite the generally accepted 97% sequence similarity cut-off value for the recognition of species (Ryberg 2015), they still hesitated to describe them as a new species. More recently, there has been a considerable increase of new *Russula* species described from Southeast Asia. For example, 24 new *Russula* species have been described from China in the period 2005–16 (Li et al. 2016), and an additional eight new species have been recognized there in 2017, which indicates an increasing annual rate (Das et al. 2017a, b). However, only two new *Russula* taxa have so far been described from higher elevations of the montane belt (2500–4000 m a.s.l.) of the southeast Tibetan Plateau: *R. wangii* and *R. amethystina* subsp. *tengii* (Li et al. 2016). Accordingly, in the area of the southeast Himalayas, most taxa have been described from lower elevations of up to 2300 m (Joshi et al. 2012), and only *R. shingbaensis* and *R. thindii* have been found in subalpine coniferous forests (elevations above 3000 m) with *Abies densa* (Das et al. 2014).

The montane belt of the Himalayan Mountains has developed an autonomous flora differing from Eurasian boreal taiga forests because of isolation by distance and climatic disjunctions (Miehe et al. 2015). The montane coniferous forests that flank the Himalayas at their southeast to southwest edge are not connected to the Siberian boreal taiga forests. The closest areas with a connection to Eurasian taiga forests are the Tian Shan and Altai Mountains to the northwest and Inner Mongolia to the northeast. Singer (1938) reported 33 *Russula* taxa from the montane, subalpine to alpine belts of the Altai and four *Russula* species from similar areas of Tian Shan. To his Asiatic collections from the area, he assigned 21 European *Russula* names and described three new species, five new subspecies, four new varieties and one new form. His alpine species *R. oreina* is considered a synonym of the European *R. pascua* (Knudsen & Borgen 1992a) and his *R. mesospora* (associated with *Betula*) a synonym of the European *R. intermedia* (Ruotsalainen & Vauras 1994). *Russula citrinochlora*, described from the subalpine belt of the Altai Mountains, has also been reported from Greenland (Knudsen & Borgen 1992b) and Fennoscandia (Marstad 2004). Only a few *Russula* species have been reported from Inner Mongolia (e.g. Tian et al. 2014) and only one, *R. jilinensis* (Li et al. 2012), has been described as new from the region.

The assembly of phylogenetic community structure and species pool scaling of forest communities in East Asia revealed the important role of intercontinental migration during the Neogene and Quaternary in the formation of species diversity in the area (Feng et al. 2015). Study of the global biogeographic distribution of *Alnus*-associated ECM fungi (Põlme et al. 2013) indicated co-dispersal of hosts and their mycobionts, but at a regional scale, geographic distance or disjunctions may also largely account for the observed diversity (Bahram et al. 2012).

In this study we attempted to test the hypothesis that geographic distance, a host switch or a climatic disjunction contribute to the evolution of ECM fungi using the *Russula globispora* lineage. This lineage belongs to the subsection *Maculatinae* and is morphologically defined by a slightly acrid taste of the flesh, yellow spore print, yellow-brownish spots on the basidiomata and spores with isolated large warts or spines (Adamčík & Jančovičová 2013, Adamčík et al. 2016b). In the *R. globispora* lineage, *R. globispora* and *R. dryadicola* are the only species reported from boreal, arctic or alpine habitats of Europe (Sarnari 1998). The aim of this study was to analyse phylogenetic relationships among collections and available sequence data of the *R. globispora* lineage originating from coniferous subtropical, mixed boreal and alpine forests of Europe, the southeast Himalayas, and North America.

## MATERIAL AND METHODS

### Sampling

We analysed 10 collections of *Russula globispora* and *R. dryadicola* from various habitats across Europe, including temperate deciduous forests, boreal mixed forests and alpine habitats. Asian material from the south Himalayan Mountains is represented by four morphologically similar collections from subtropical coniferous rainforests of *Pinus roxburghii* in Pakistan, and four other similar collections from montane mixed forests of China associated with *Abies spectabilis* and *Betula delavayi*. Three representatives of subsection *Cupreinae*, identified and sequenced by us, were used as the outgroup. This study is supplemented by sequences with high (99%) similarity retrieved from the Genbank (https://www.ncbi.nlm.nih.gov) and UNITE (https://unite.ut.ee) databases. All samples and sequences used are listed in Additional file 1: Table S1.

### Molecular analysis

Total genomic DNA was extracted from dried material using a variety of protocols (Eberhardt 2012, Adamčík et al. 2016a). In addition to these protocols, the EZNA Fungal DNA Mini Kit (Omega) was used following the manufacturer’s recommendations, but with prolonged incubation time of up to 1 h after addition of the RNA-lytic enzyme. Three molecular markers were amplified: (1) the internal transcribed spacer regions of ribosomal DNA (ITS); (2) the partial mitochondrial small subunit of ribosomal DNA (mtSSU); and (3) the region between domains six and seven of the nuclear gene encoding the second largest subunit of RNA polymerase II (*rpb2*). The ITS region was amplified using the primers ITS1F–ITS4 (White et al. 1990, Gardes & Bruns 1993). The mtSSU region was amplified using the primer pair MS1 and MS2 (White et al. 1990). The *rpb2* was amplified using the primers A-Russ-F–frpb2-7CR (Matheny 2005, Caboň et al. 2017). All three molecular markers were amplified with Hot Start Firepol Polymerase (Solis Biodyne, Tartu, Estonia) using cycling protocols according to Caboň et al. (2017). The PCR products were purified using Exo-Sap enzymes (Thermo Fisher Scientific, Wilmington, DE) or the Qiaquick PCR Purification Kit (Qiagen, Hilden, Germany). Samples were sequenced by the Seqme company (Dobříš, Czech Republic).

### Phylogenetic analysis

Raw sequences were edited in Geneious version R10 (Kearse et al. 2012). Intra-individual polymorphic sites having more than one signal were marked with NC-IUPAC ambiguity codes. All three single-locus datasets were aligned by MAFFT version 7 using the strategy E-INS-i (Katoh & Standley 2013), manually improved in Geneious version R10 (Kearse et al. 2012) and concatenated into one multi-locus dataset using SeaView version 4.5.1 (Gouy et al. 2010). The multi-locus dataset was analysed using two different methods: Bayesian inference (BI) and the maximum likelihood (ML) approach. For the ML analysis, the concatenated alignment was loaded as a *PHYLIP* file into RAxMLGUI v. 1.2 (Silvestro & Michalak 2012) and analysed as a partitioned dataset under the GTR + I model with 1000 bootstrap iterations. In addition, the GTR + G model was used as recommended by Stamatakis (2008). Phylogenetic analyses generated using both models resulted in trees with identical topology and very similar bootstrap support. Thus, only the GTR + G + I analysis is presented. For the BI analysis, the dataset was divided into six partitions: ITS, mtSSU, intronic region 7 of *rpb2*, and the 1st, 2nd and 3rd codon positions of *rpb2*. The best substitution model for each partition was computed jointly in PartitionFinder v. 1.1.1 (Lanfear et al. 2012). The BI was computed independently twice in MrBayes version 3.2.6 (Ronquist et al. 2012) with four MCMC chains for 10,000,000 iterations until the standard deviation of split frequencies fell below the 0.01 threshold. The convergence of runs was visually assessed using the Trace function in Tracer version 1.6 (Rambaut et al. 2013).

### Morphological analysis

Micromorphological characteristics were observed using an Olympus CX-43 microscope with an oil-immersion lens at a magnification of 1000×. All drawings of microscopic structures, with the exception of spores, were made with a ‘camera lucida’ using an Olympus U-DA drawing attachment at a projection scale of 2000×. The contents of hymenial cystidia and pileocystidia were illustrated as observed in Congo red preparations from dried material, with the exception of some pileocystidia for which the contents are indicated schematically (by plus signs). Spores were observed on the lamellae stained with Melzer’s reagent. All other microscopic observations were made in ammoniacal Congo red after a short treatment in a warm aqueous KOH solution to dissolve the gelatinous matrix and improve tissue dissociation. All tissues were also examined in Cresyl blue to verify the presence of ortho- or metachromatic reactions as explained in Buyck (1989). The trama and cystidia were examined in a sulfovanillin solution. Acidoresistant incrustations of primordial hyphae were stained with carbolfuchsin and observed in distilled water after incubation for a few seconds in a 10% solution of HCl (cf. Romagnesi 1967). Spores were scanned with an Artray Artcam 300MI camera and measured by Quick Micro Photo version 2.1 software with an accuracy of 0.1 μm. Spore measurements excluded ornamentation and their line drawings were made using enlarged, scanned pictures. The Q value indicates the length/width ratio of the spores. Spore ornamentation density was estimated following Adamčík & Marhold (2000). Estimates of the density of cystidia estimates follow Buyck (1991). Statistics for the measurements of microscopic characteristics were based on 30 measurements per specimen and are based on all examined material of the described species. The range of measured values is expressed as the mean ± standard deviation; presented in parentheses are the 5th and 95th percentiles.

## RESULTS

### Phylogenetic analysis

**Fig. 1:**
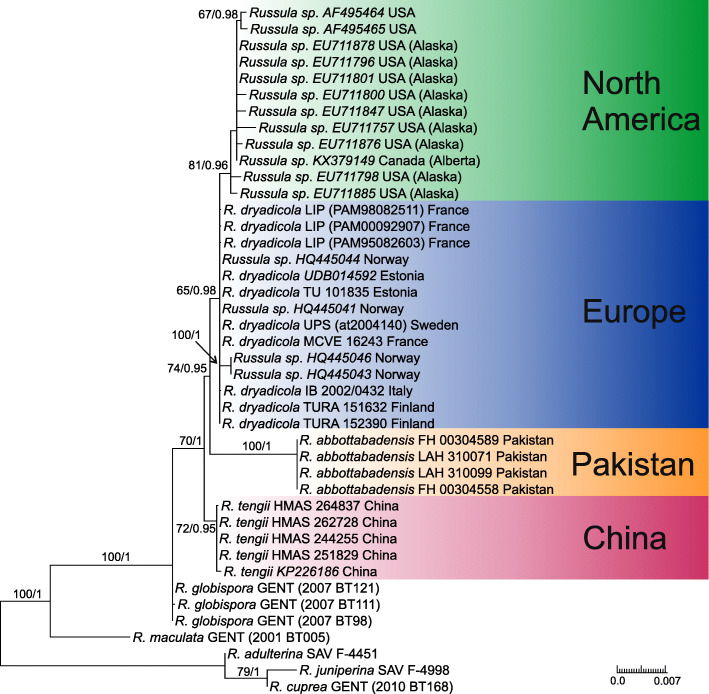
Phylogram generated by Maximum Likelihood (RAxML) analysis based on combined sequence data of ITS, mtSSU and *rpb2* for five species of *Russula* subsect. *Maculatinae* and three outgroup sequences. Maximum likelihood bootstrap support values greater than 50% and Bayesian posterior probabilities greater or equal to 0.90 are indicated above or below the nodes. More details about all sequences used in this study are presented in Additional file 1: Table S1

In total, 54 sequences were newly generated and the final dataset was supplemented by 28 published sequences, including 20 ITS sequences with high sequence similarity to *Russula dryadicola* retrieved from public databases (Additional file 1: Table S1). They altogether correspond to 42 collections in the tree (Fig. 1), and 38 of them are placed in the *R. globispora* clade with full support. Three collections of *R. globispora* from temperate deciduous forests are placed at the basal position of the clade. Collections from all other habitats (coniferous and boreal forests or arctic-alpine habitats) are clustered in a clade with moderate ML and full BI support (70/1). This clade is composed of three well supported clades with various branch length, each corresponding to samples from different habitats and geographic areas. Our collections from *Pinus roxburghii* mono-dominant forests of the southwest Himalayan Mountains (Pakistan) are grouped in a fully supported clade and below we describe them as a new species bearing the name *R. abbottabadensis*. Another well supported species clade is formed by collections from mixed montane forests of the southeast Himalayas (China) and corresponds to the second new species, *R. tengii*, described below. The third clade groups samples that originated from a large geographic area of Europe and Alaska, collected in boreal, arctic and alpine habitats. Alaskan samples retrieved from the Genbank database received strong support (81/0.96), but they are represented only by ITS sequences and differ from other samples within this clade of European origin only in 1–2 parsimony-informative positions. All European collections correspond morphologically and ecologically to the concept of *R. dryadicola*.

### Morphological analysis

All four species in the *Russula globispora* clade are morphologically defined by yellow-brownish spots on the surface of the basidiomata, yellow spore print and relatively large spores, which are characters that correspond to the circumscription of *R.* subsect. *Maculatinae*. In addition, all studied members of the *R. globispora* clade have large isolated spines on the spores and a weakly acrid to mild taste that distinguishes them from other members of the subsection. In the field, *R. abbottabadensis* is easily recognized by the bright red colours on the pileus showing only a little discolouration in spots near the centre, whereas the other two species usually have dull pink to violet colours and are strongly discoloured near the centre. The basidiomata of *R. dryadicola* are more robust and larger.

Under the microscope, the most important differences were observed in the pileipellis. *Russula abbottabadensis* has long (on average longer than 29.5 μm), narrow (on average to 3 μm), cylindrical terminal cells of hyphae in the pileipellis near the pileus margin with usually branched subterminal cells and narrower (on average to 5 μm), prevailingly 1–2-celled pileocystidia. *Russula tengii* has shorter (on average to 29.5 μm) terminal cells of hyphae in the pileipellis near the pileus margin, less branched subterminal cells, and wider (on average more than 5 μm) and more septate (mainly 2- or more-celled) pileocystidia. *Russula dryadicola* is well characterized by wider (on average more than 3.5 μm), frequently fusiform or lanceolate terminal cells of hyphae in the pileipellis near the pileus margin that are often moniliform.

There are no other species known from southeast Himalayas that remind morphologically or in sequence *R. globispora* lineage. However, according to our knowledge (K. Wisitrassameewong, personal communication), there are probably more undescribed members of the lineage from South Korea and possibly other areas of Southeast Asia. Our detailed *Russula* descriptions based on robust statistical support of several measurements from multiple collections will undoubtedly serve as a good base for their morphological delimitation and support the phylogeny based species hypothesis.

## TAXONOMY

**Fig. 2:**
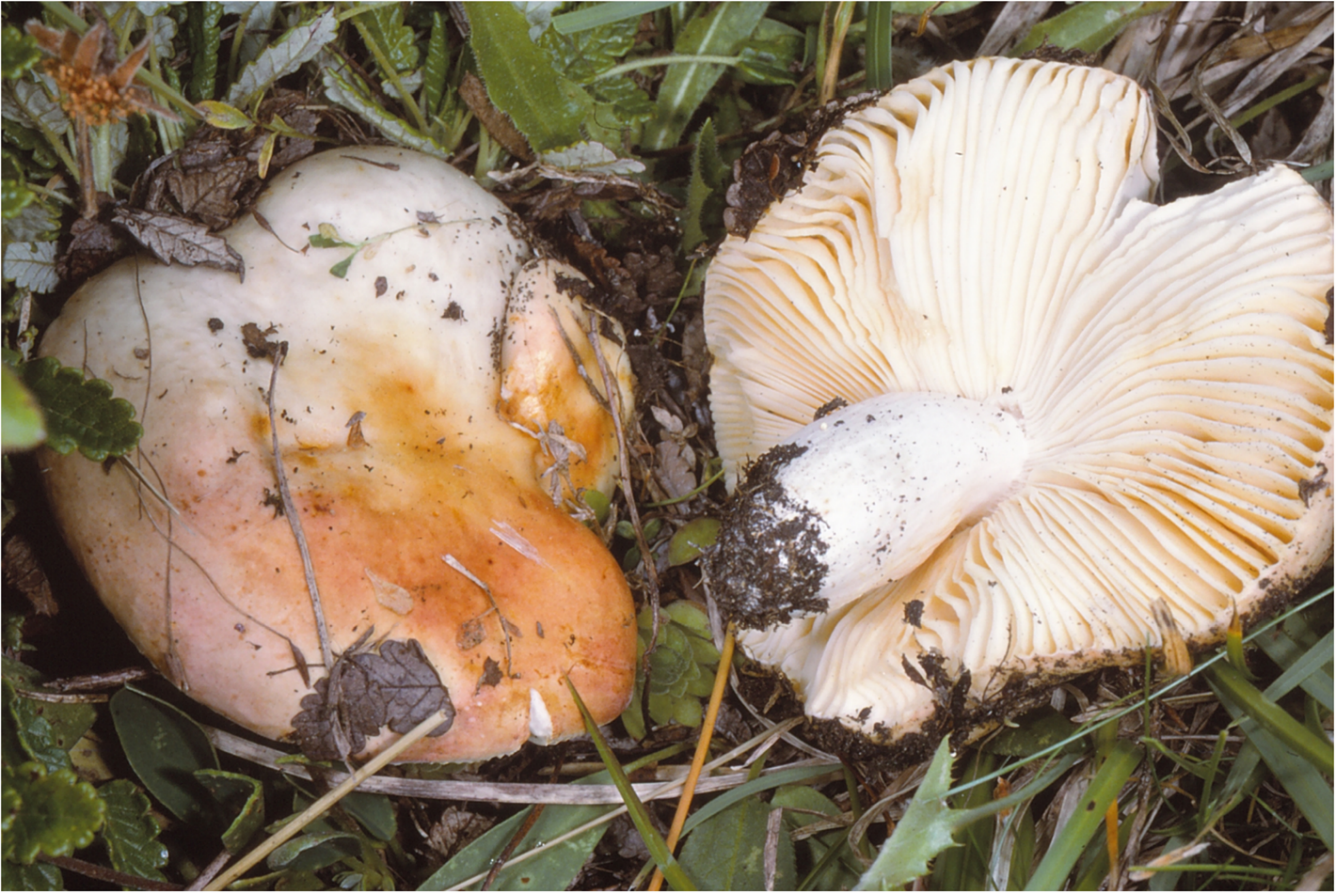
*Russula dryadicola* (LIP PAM95082603), field appearance. Bar = 1 cm. Photo: P.-A. M

**Fig. 3:**
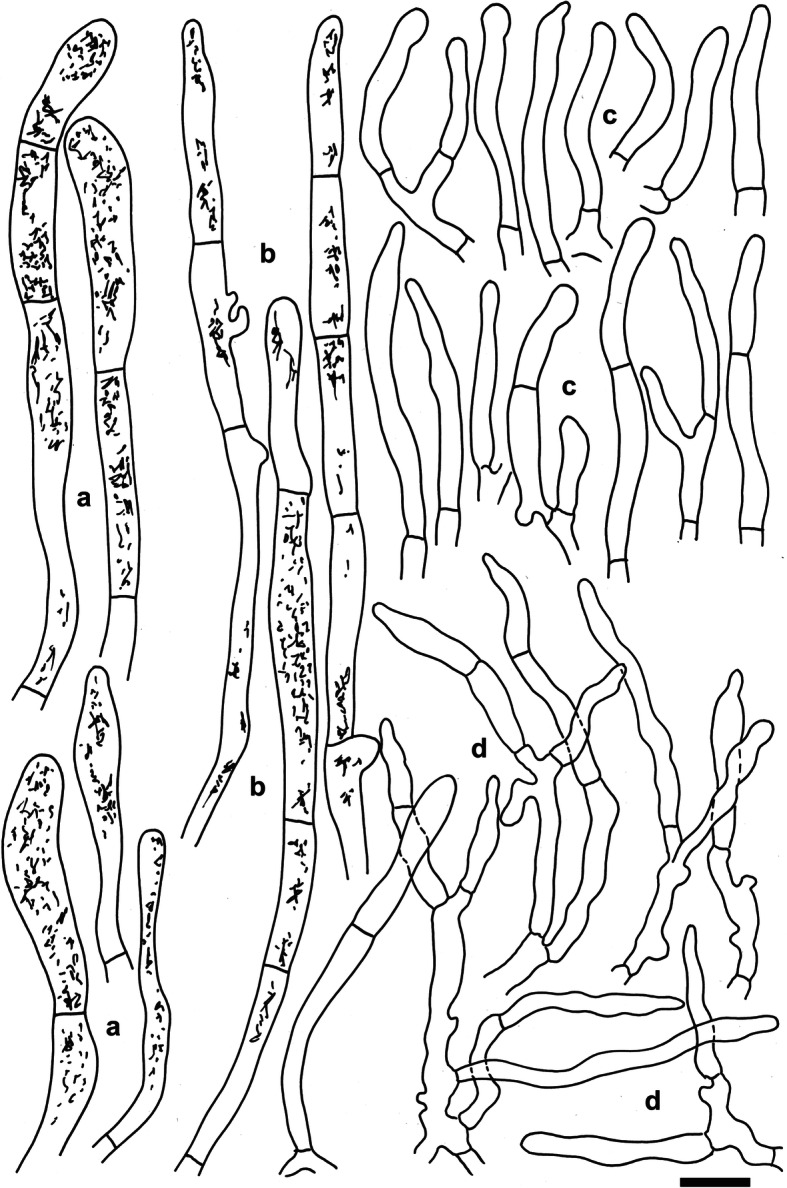
*Russula dryadicola* (IB 1997/0750, neotype). Microscopic features of the pileipellis. **a**. Pileocystidia near the pileus centre. **b**. Pileocystidia near the pileus margin. **c**. Hyphal terminations in the pileus centre. **d.** Hyphal terminations near the pileus margin. Contents of cystidia are as observed in Congo red. Bars = 10 μm. Drawings: S.A. & S.J

**Fig. 4:**
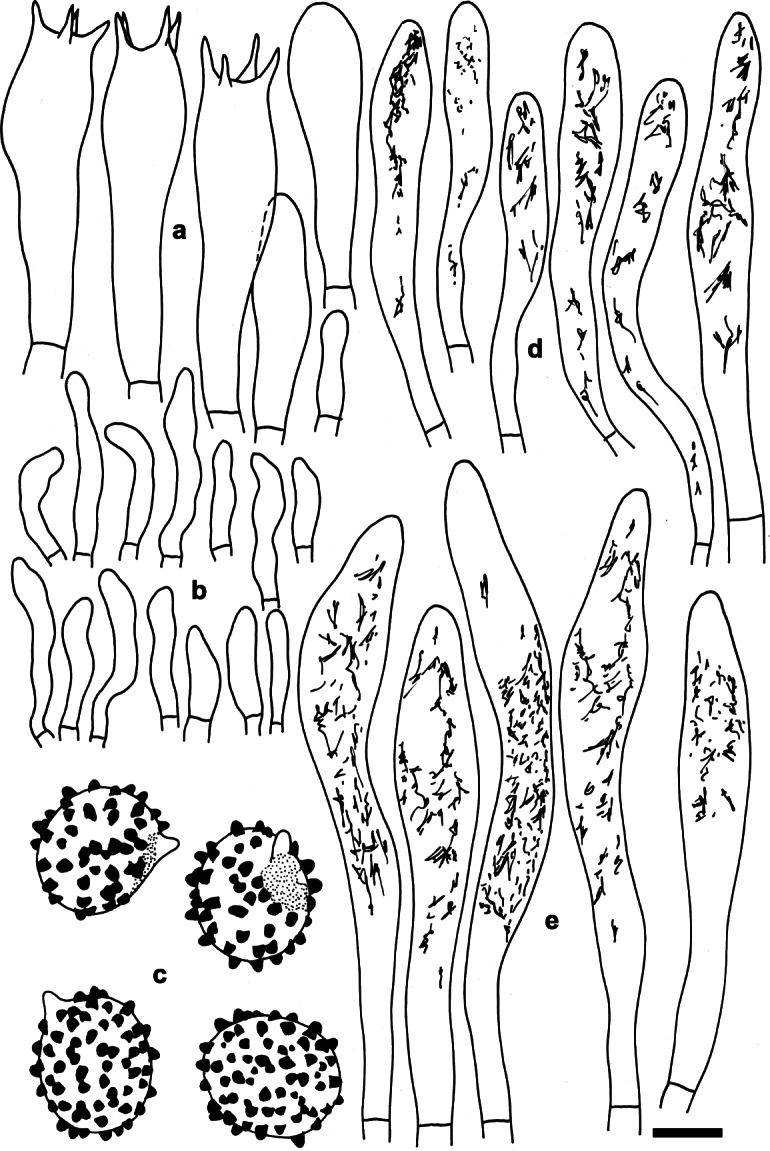
*Russula dryadicola* (IB 1997/0750, neotype). Microscopic features of the hymenium. **a.** Basidia and basidiola. **b.** Marginal cells. **c.** Basidiospores in Melzer’s reagent. **d.** Cheilocystidia. **e.** Pleurocystidia. Contents of cystidia are represented as observed in Congo red. Bars = 10 μm, spores = 5 μm. Drawings: S.A. & S.J

**Russula dryadicola** Singer ex R. Fellner & Landa, *Biblioth. Mycol*. **150**: 34 (1993); Figs. 2, 3, 4.

*Type*: **Switzerland**: Ticino, Olivone, Lucomagno, alt. 1950 m, among *Dryas* sp. and *Juniperus* on limestone, 15 August 1995, *G. Lucchini* (IB 1997/0750 – neotype, designated by Sarnari 1998).

*Description: Basidiomata* medium-sized but robust. *Pileus* 40–130 mm diam, first hemispherical, soon applanate, with age slightly depressed; margin never striated or tuberculate; pileus cuticle thick, hardly separable, sometimes slightly rugulose, greasy when moist, then shiny; colour first uniformly whitish, soon becoming salmon-orange near the margin, when mature sometimes with copper-red, pink-reddish ‘vesca’, or even purplish red tints irregularly developing near the centre, under sun exposure usually remaining pale, butter yellow to golden yellow, irregularly rusty yellow spotted. *Lamellae* rather distant, *ca* 60 reaching the stipe, adnexed, often forming a collar near the stipe, broad, ventricose, occasionally forked, first pale cream, when mature light yellow; edge even, concolourous. *Stipe* 40–70 × 15–25 mm, central or slightly eccentric, cylindrical to fusiform, often curved, solid, rugulose; white, with early-developing rusty spots at the base, when old slightly yellowing throughout. *Context* firm, softer in the stipe, white, slowly turning yellow-brownish when wounded. *Smell* rather strong, of hot bread, somewhat honey-like at the stipe base. *Taste* mild to slightly acrid. *Spore print* yellow (IVa at the scale of Romagnesi 1967 according to Biscoletto & Ostellari 2005).

*Spores* (7.9–)8.7–9.5–10.2(− 11) × (6.9–)7.6–8.2–8.8(− 9.5) μm, subglobose to broadly ellipsoid, Q = (1.08–)1.12–1.16–1.23(− 1.29), ornamentation of large, distant [(2–)3–5(− 6) in a 3 μm diam circle] amyloid warts, (0.7–)0.8–1(− 1.1) μm high; occasionally fused in pairs [0–1(− 2) fusions in the circle], line connections very rare and short (0–1 in the circle), warts mainly isolated; suprahilar plage large, amyloid. *Basidia* (41–)48.5–54–59(− 65) × (12.5–)13–13.9–15(− 16) μm, 4-spored, clavate, pedicellate; basidiola first cylindrical, then clavate, ca 4–12 μm wide. *Subhymenium* pseudoparenchymatic. *Lamellar trama* mainly composed of large sphaerocytes. *Pleurocystidia* dispersed, 450–700/mm^2^, (70–)75.5–86.2–97(− 130) × (9–)10.5–11.6–12.5(− 14.5) μm, fusiform or clavate, pedicellate, apically acute to subacute, without or with 5–10(− 24) μm long appendage, thin-walled or occasionally with slightly thickened walls (to 0.75 μm thick), contents heteromorphous, banded, slowly turning red-brown in sulfovanillin. *Cheilocystidia* abundant, usually protrude more than half of their length above densely arranged marginal cells, smaller than pleurocystidia, (39–)46–55.5–65(− 85) × (5.5–)6.5–7.7–9(− 10.5) μm, usually clavate, occasionally fusiform, pedicellate, apically mainly obtuse, contents usually banded and yellow-pigmented. *Marginal cells* smaller and narrower than basidiola on gill sides, subcylindrical, flexuous, often also slightly moniliform, occasionally nodulous, (14–)20–26.3–32.5(− 45) × (3–)4.5–5.3–6.5(− 7.5) μm, apically obtuse or subacute. *Pileipellis* orthochromatic in Cresyl blue, sharply delimited from the underlying sphaerocytes of the context, strongly gelatinized in all parts, 140–150 μm deep, vaguely divided in a 65–90 μm deep suprapellis of ascending or erect, but near the surface often repent, relatively densely packed hyphae, gradually transitioning into a dense, 65–80 μm deep subpellis of intricate, irregularly oriented, but near the trama horizontally oriented, 2.5–5(− 10) μm wide hyphae. Acid-resistant incrustations absent, but the contents of pileocystidia stain red after carbol fuchsin treatment. Hyphal terminations near the pileus margin slender, distinctly moniliform, often slightly flexuous, thin-walled, with terminal cells (12.5–)18–25.6–33(− 40) × (2.5–)3–3.8–4.5(− 5) μm, mainly cylindrical, occasionally subulate or lanceolate, apically usually constricted to ca 1.5–2.5 μm, but not attenuated; subterminal cells branched or not, usually equally wide and long, often with lateral nodules and rarely also with lateral branches. Hyphal terminations near the pileus centre less moniliform and usually cylindrical, with terminal cells (12–)18–25.3–33(− 40) × 2.5–3.4–4(− 5) μm, apically obtuse and rarely constricted. *Pileocystidia* near the pileus margin numerous, 1–1.9–3 celled, thin-walled, terminal cells (19–)28–54–80(− 106) × (4–)4.5–5.7–6.5(− 7.5) μm, cylindrical, obtuse, contents heteromorphous, granular or banded, often with yellow refringent pigments, hardly react in sulfovanillin, near the pileus centre with often clavate and wider terminal cells, (21–)25–36.1–47.5(− 58.5) × (4–)4.5–6.1–8(− 9) μm; subterminal cells usually equally large, occasionally longer or wider. Cystidioid hyphae observed only in the upper part of the subpellis, absent in the pileus trama. *Clamp connections* absent in all parts.

*Additional material examined*: **Italy**: *Südtirol*: Sexten, Fischleintal, near Zsigmondyhütte, alt. *ca* 2400 m, 5 Aug. 2006, *U. Peintner & I. Göschl* (IB 2002/0432). **– Finland**: *Enontekion Lappi*: Kilpisjärvi, Saana, near the biological station, mountain birch forest, 16 Aug. 1990, *J. Ruotsalainen* (TURA 151632); Etelä-Häme, Raikonkulma, Raikko, E of Kivijärvi lake, Kalkkimäki, herb rich forest with *Picea*, *Pinus*, *Betula*, *Salix caprea*, *Populus tremula* on calcareous soil, 22 Aug. 2003, *J. Vauras JV20125* (TURA152390). **– France**: *Savoie*: Bourg-Saint-Maurice, Arc 2000, vers col. des Frettes, alpine pasture with *Dryas octopetala*, on dolomite, 25 Aug. 1998, *P.-A. Moreau PAM98082511* (LIP); ibid., 29 Aug. 2000, *P.-A. Moreau PAM00082907* (LIP); ibid., 26 Aug. 1995, *P.-A. Moreau PAM95082603* (LIP); ibid., 4 Sept. 1994, *P.-A. Moreau PAM94090402* (LIP). **– Sweden**: Uppsala, Nåsten, 23 Sept. 2004, *A. Taylor AT2004140* (UPS).

**Fig. 5:**
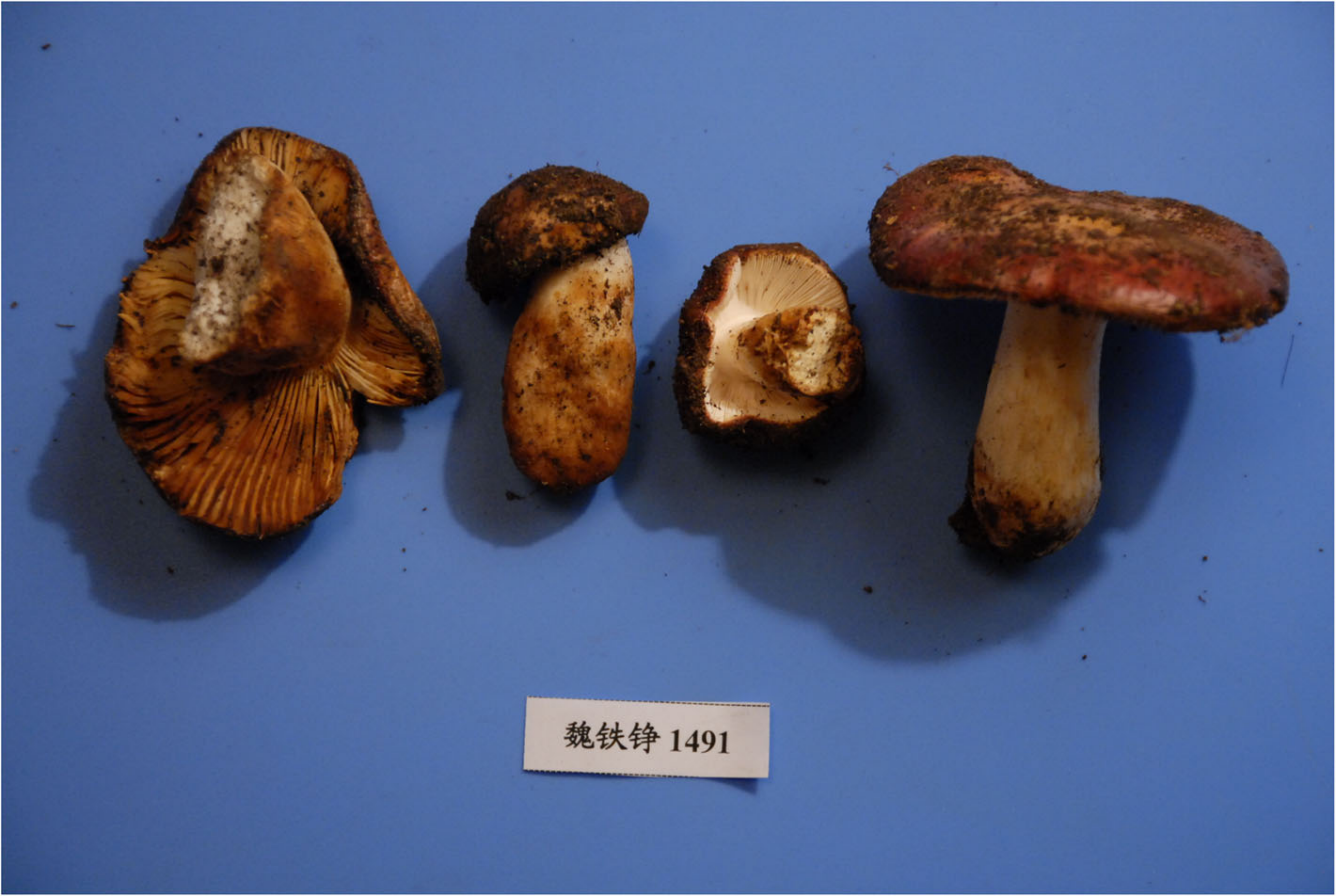
*Russula tengii* (HMAS262728, holotype), field appearance. Bar = 1 cm. Photo: G.-J.L.

**Fig. 6:**
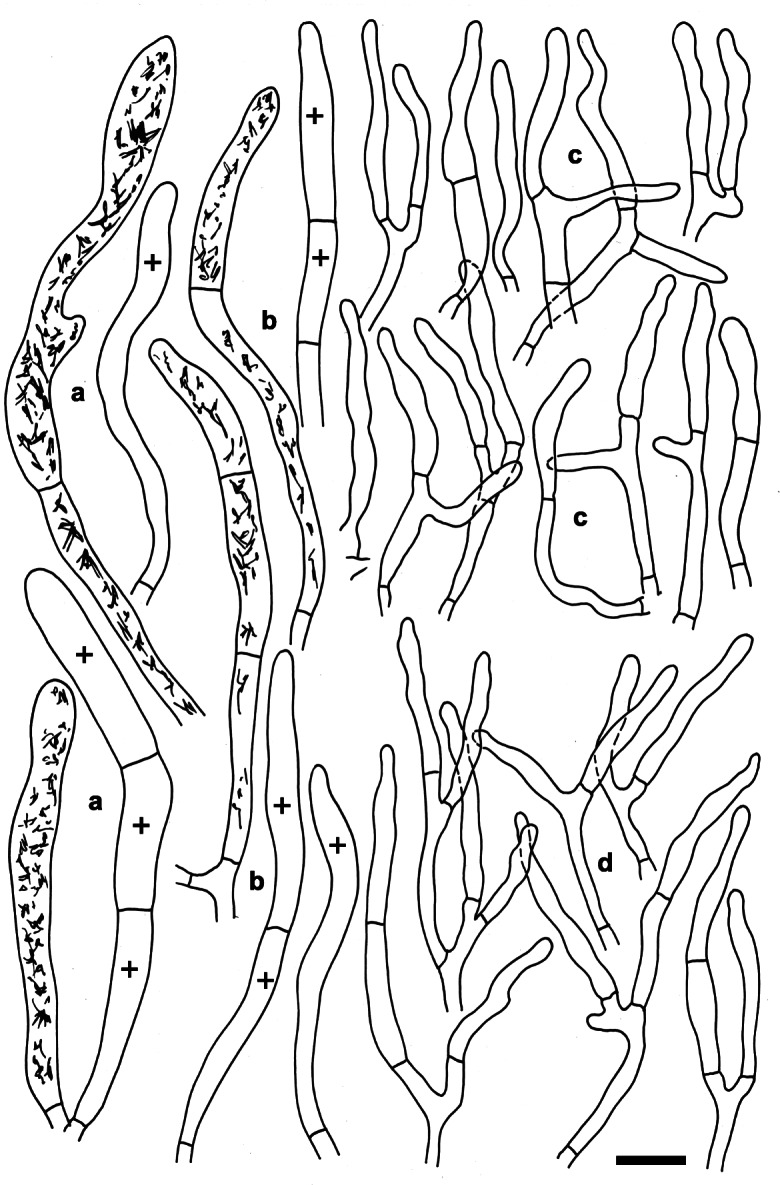
*Russula tengii* (HMAS262728, holotype). Microscopic features of the pileipellis. **a.** Pileocystidia near the pileus centre. **b.** Pileocystidia near the pileus margin. **c.** Hyphal terminations in the pileus centre. **d.** Hyphal terminations near the pileus margin. Contents of cystidia are represented as observed in Congo red. Bars = 10 μm. Drawings: S.A. & S.J

**Fig. 7:**
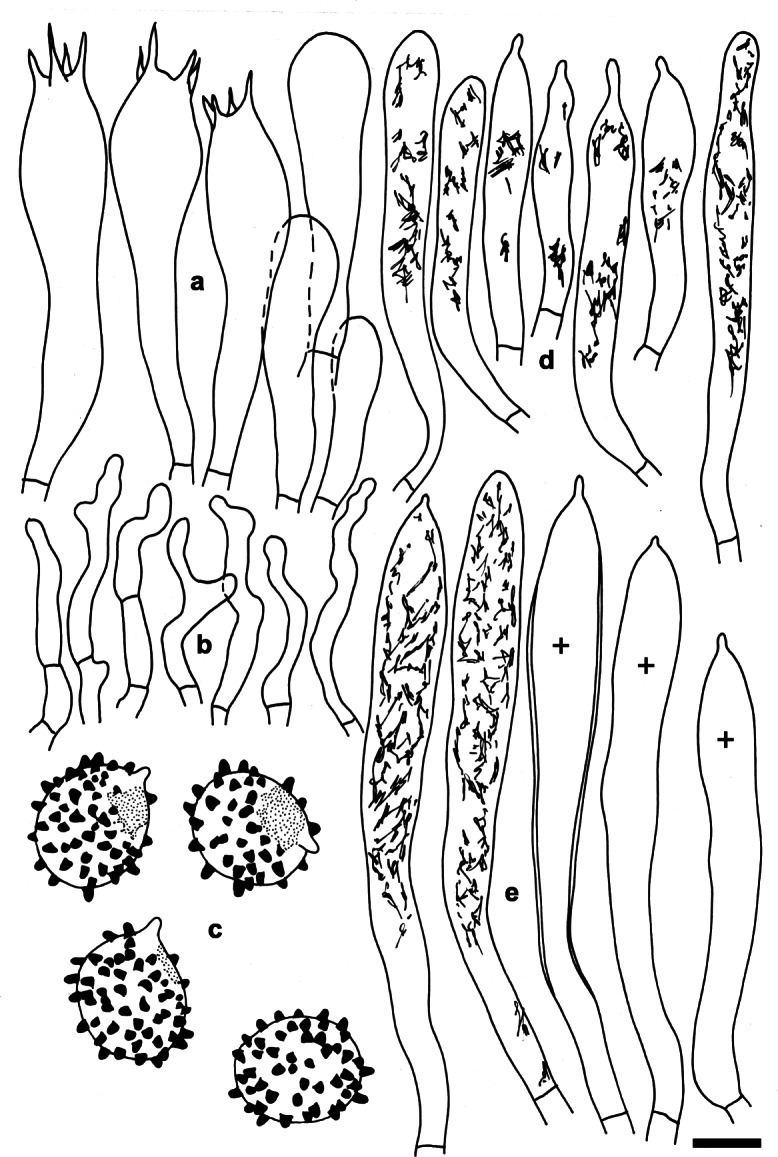
*Russula tengii* (HMAS262728, holotype). Microscopic features of the hymenium. **a.** Basidia and basidiola. **b.** Marginal cells. **c.** Basidiospores in Melzer’s reagent. **d.** Cheilocystidia. **e.** Pleurocystidia. Contents of cystidia are represented as observed in Congo red; crosses schematically indicate the contents of some cystidia. Bars = 10 μm, spores = 5 μm. Drawings: S.A. & S.J

**Russula tengii** G.J. Li & H.A. Wen, **sp. nov;** Figs. 5, 6, 7.

MycoBank No.: MB823538.

*Etymology*: Named in honour of Shu Chün Teng (1902–70) for his contribution to mycology.

*Diagnosis:* Basidiomata small to medium sized, thick-fleshed, similar to *R. dryadicola* in pileus colour and presence of yellow-brownish spots on surface of pileus and stipe, from which differs by mainly cylindrical, apically obtuse and not constricted, in average up to 3.5 μm wide hyphal terminations in pileipellis near the pileus margin and pileocystidia that are mainly two and more celled.

*Type*: **China**: *Xizang Autonomous Region*: Riwoqe County, 31°12′ N 96°36′ E, alt. 3741 m, 24 July 2010, *T.Z. Wei 1491* (HMAS262728 – holotype).

*Description: Basidiomata* small to medium-sized, robust. *Pileus* 28–78 mm diam, first hemispherical, then convex to applanate, sometimes slightly depressed; margin first slightly deflexed, when mature straight to slightly inflexed, sometimes undulate, indistinctly striate; pileus cuticle slightly viscous when wet; colour near the pileus margin light brownish vinaceous to light russet vinaceous, when old brick-red, and madder-brown when dry, near the pileus centre mustard-yellow to aniline-yellow when old and dry, variegated with dark blackish red and dark yellow spots. *Lamellae* relatively dense, 10–16 per cm near the pileus margin, adnate-emarginated, 4–6 mm broad, occasionally forked near the pileus margin, rarely near the stipe, slightly anastomosed, without lamellulae, first creamy to yellowish, when mature baryta yellow to buff yellow, pale yellow-orange when dry; edge even, concolourous. *Stipe* 35–47 × 12–15 mm, central or slightly eccentric, cylindrical, often swollen toward the base, solid to hollow when mature, smooth; white, soon with a buff yellow tint, especially near the base. *Context* 3–7 mm thick at the attachment of lamellae to the stipe, compact but fragile in gills, white, slightly turning pale creamy or yellowish when wounded or old. *Smell* indistinct. *Taste* mild. *Spore print* yellow (IVc–IVd according to Romagnesi 1967).

*Spores* (8.1–)8.9–9.7–10.5(− 11.3) × (6.9–)7.5–8.2–8.9(− 9.5) μm, broadly ellipsoid, Q = (1.14–)1.15–1.18–1.21(− 1.24), ornamentation of large, moderately distant [(3–)4–6(− 7) in a 3 μm diam circle] amyloid warts, (0.6–)0.8–1(− 1.2) μm high; occasionally fused in pairs [0–2(− 3) fusions in the circle], line connections very rare and short [0–2(− 3) in the circle], warts mainly isolated; suprahilar plage large, amyloid. *Basidia* (51–)54–58.1–62(− 66) × (13–)13.5–14.7–15.5(− 16.5) μm, 4-spored, clavate, pedicellate; basidiola first cylindrical, then clavate, ca 5–13 μm wide. *Subhymenium* pseudoparenchymatic. *Lamellar trama* mainly composed of large sphaerocytes. *Pleurocystidia* dispersed to moderately numerous, ca 600–800/mm^2^, (73–)79.5–88.7–98(− 112) × (9–)10–11.1–12.5(− 14.5) μm, fusiform or clavate, usually pedicellate, apically subacute to obtuse, mainly with 1–8(− 13) μm long appendage, usually with slightly thickened walls (*ca* 0.5 μm thick), contents heteromorphous, banded, slowly turning red-brown in sulfovanillin. *Cheilocystidia* abundant, (39–)44.5–54.5–65(− 81) × (5–)6.5–7.4–8.5(− 9.5) μm, usually clavate, occasionally fusiform, pedicellate, apically mainly obtuse, occasionally with a short (2–6 μm) appendage, few with slightly thickened walls (to 0.5 μm thick), contents usually banded, often dispersed and yellow-pigmented. *Marginal cells* smaller and narrower than basidiola on gill sides, subcylindrical, flexuous, often also slightly moniliform, occasionally nodulous, (14–)22–29.2–36.5(− 41) × (2.5–)3–3.9–4.5(− 5.5) μm, apically obtuse or slightly constricted. *Pileipellis* orthochromatic in Cresyl blue, sharply delimited from the underlying sphaerocytes of the context, strongly gelatinized in all parts, 160–200 μm deep, vaguely divided in a 55–80 μm deep suprapellis of erect but often irregularly oriented, near the surface loosely arranged hyphae, gradually transitioning into a dense, 100–130 μm deep subpellis of intricate, irregularly oriented, but near the trama horizontally oriented, (2–)2.5–5(− 6.5) μm wide hyphae. Acid-resistant incrustations absent, but the contents of pileocystidia stain red after carbol fuchsin treatment. Hyphal terminations near the pileus margin slender, distinctly moniliform, thin-walled, with terminal cells (15–)21–26.8–32.5(− 39) × 2.5–3.1–3.5(− 4) μm, mainly cylindrical, occasionally subulate, rarely lanceolate, apically usually obtuse, not frequently constricted; subterminal cells branched or not, usually equally wide and long, occasionally with lateral nodules and rarely also with lateral branches. Hyphal terminations near the pileus centre similar in shape but occasionally also distinctly clavate, with terminal cells (15–)19–24–29(− 39) × 2.5–3.4–4(− 5) μm, apically obtuse and rarely constricted. *Pileocystidia* near the pileus margin numerous, 1–2.5–4(− 5) celled, thin-walled, terminal cells (18–)24.5–42.7–61(− 79) × 4.5–5.4–6(− 7) μm, cylindrical, obtuse or apically slightly constricted, contents heteromorphous, granular or banded, often with yellow refringent pigments, in sulfovanillin turning slowly to grey-brown; near the pileus centre with often clavate and wider terminal cells, (15–)18–36.3–54(− 71) × (4–)5–6.4–8(− 9) μm, apically obtuse to rounded; subterminal cells usually equally large, occasionally longer, rarely wider. Cystidioid hyphae with heteromorphous contents and yellowish pigments present in the subpellis, dispersed or absent in the pileus trama. *Clamp connections* absent in all parts.

*Additional material examined*: **China**: *Xizang Autonomous Region*: Maizhokunggar County, Riduo Township, 29°38′ N 92°26′ E, *Abies*-*Betula* forest, 6 Aug. 2012, *W.L. Lu 100* (HMAS244255); ibid., Gesang village, 29°48′ N, 92°39′ E, *Abies*-*Betula* forest, 16 Aug. 2012, *G.J. Li, D. Zhao & W. Li 12,163* (HMAS251829); ibid., 16 Aug. 2012, *G.J. Li, D. Zhao & S. Qi 12,358* (HMAS264837).

**Fig. 8:**
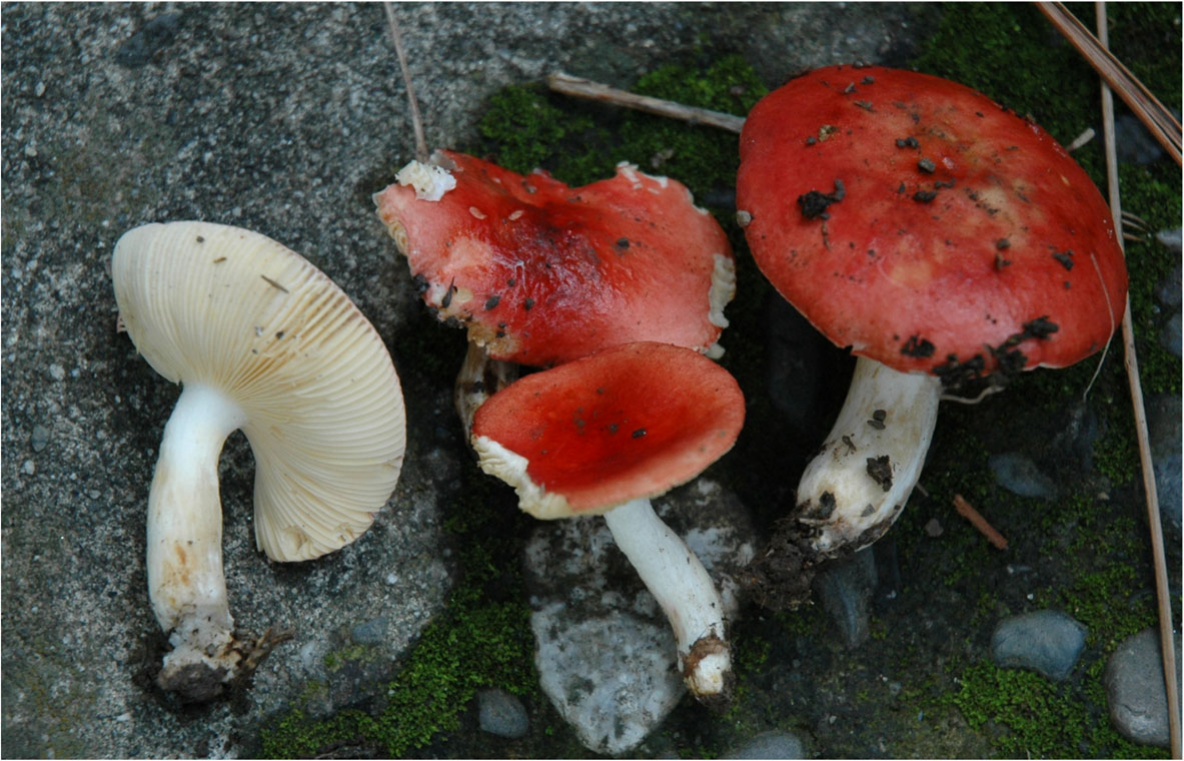
*Russula abbottabadensis* (FH00304589, holotype), field appearance. Bar = 1 cm. Photo: M.S.

**Fig. 9:**
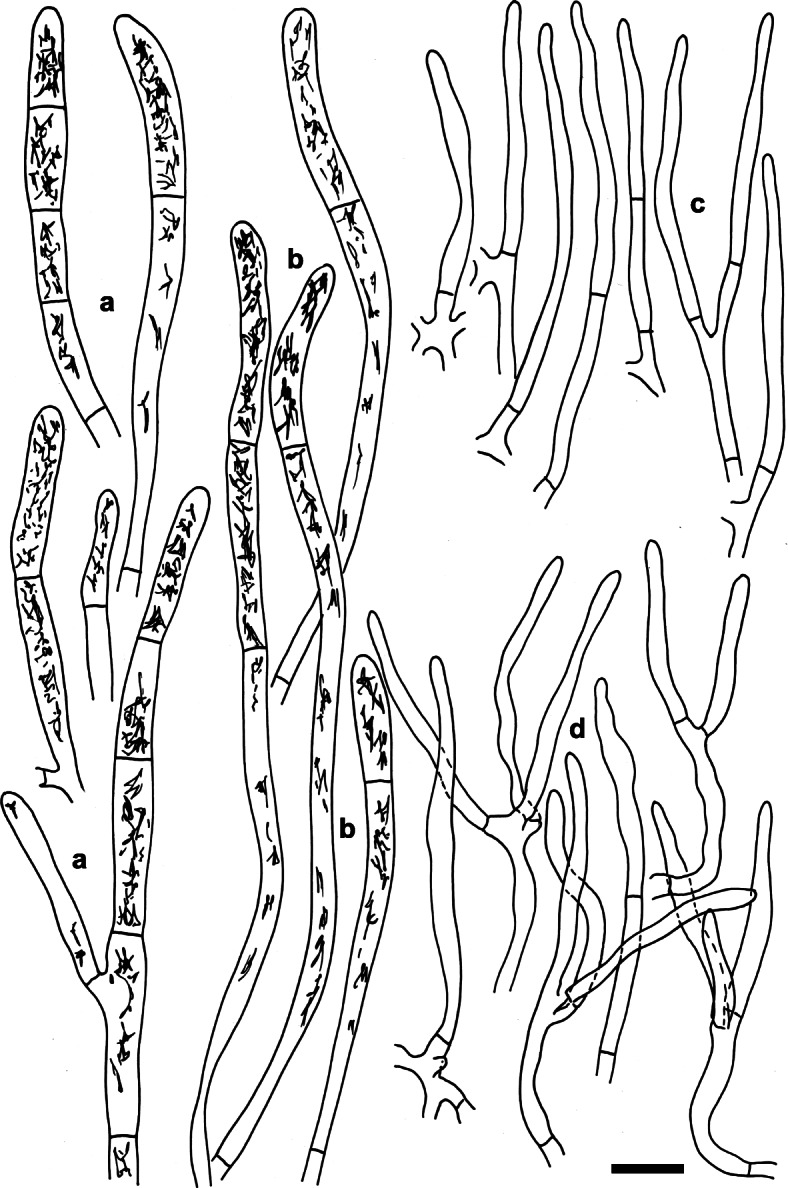
*Russula abbottabadensis* (FH00304589, holotype). Microscopic features of the pileipellis. **a.** Pileocystidia near the pileus centre. **b.** Pileocystidia near the pileus margin. **c.** Hyphal terminations in the pileus centre. **d.** Hyphal terminations near the pileus margin. Contents of cystidia are represented as observed in Congo red. Bars = 10 μm. Drawings: S.A. & S.J

**Fig. 10:**
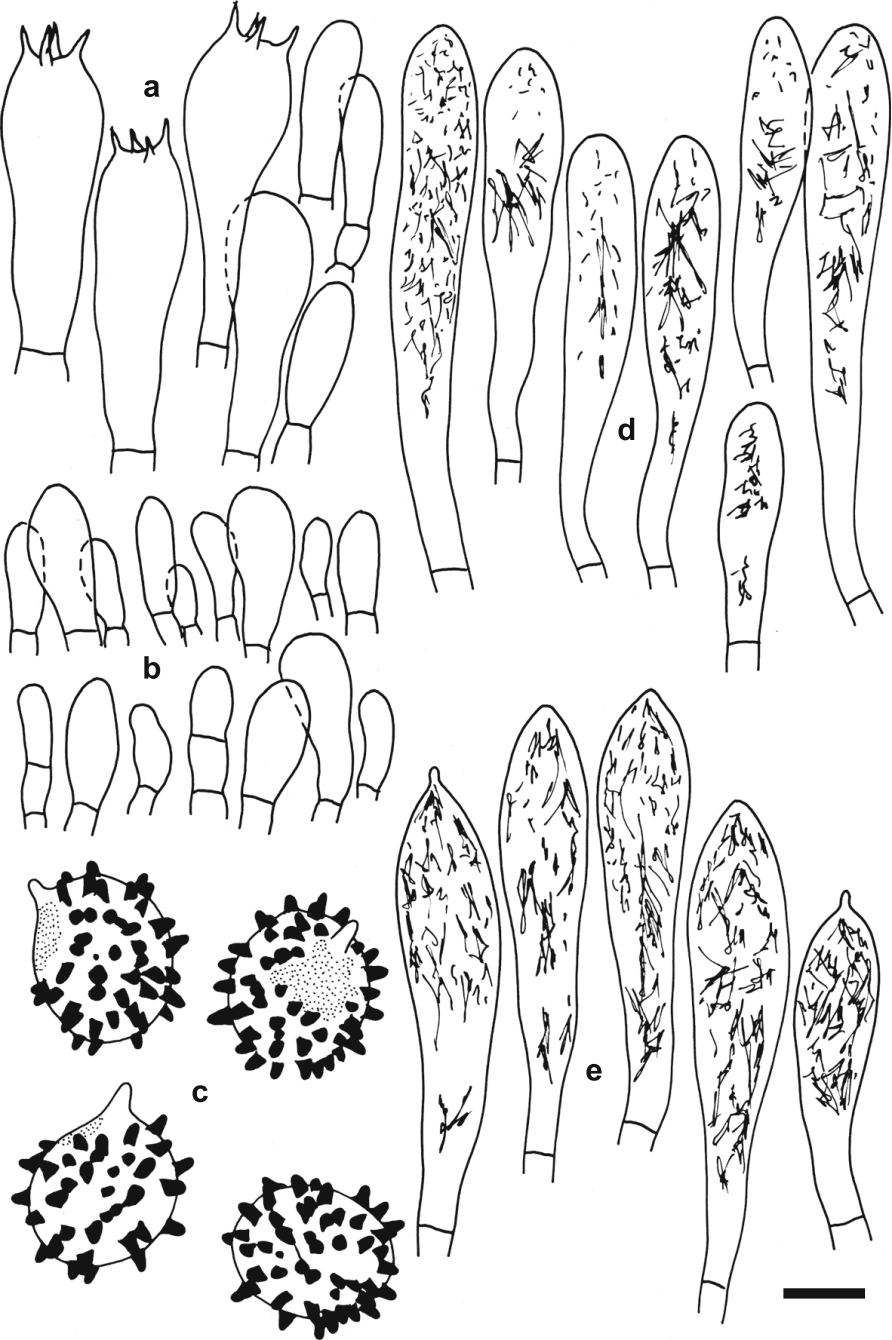
*Russula abbottabadensis* (FH00304589, holotype). Microscopic features of the hymenium. **a.** Basidia and basidiola. **b.** Marginal cells. **c.** Basidiospores in Melzer’s reagent. **d.** Cheilocystidia. **e.** Pleurocystidia. Contents of cystidia are represented as observed in Congo red. Bars = 10 μm, spores = 5 μm. Drawings: S.A. & S.J

**Russula abbottabadensis** Saba & Adamčík, **sp. nov;** Figs. 8, 9, 10.

MycoBank No.: MB823541;

*Etymology*: referring to the district of Abbottabad, the area of origin of the samples under study.

*Diagnosis:* Basidiomata small to medium sized, pileus red, stipe with yellowish brown rusty spots, taste mild to slightly acrid, spore print yellow, spores large, similar as in *R. globispora* but with occasionally fused spines, Hyphal terminations in pileipellis near the pileus margin with narrow (in average up to 3 μm), often attenuated terminal cells and mainly branched subterminal cells, pileocystidia narrow (in average up to 5 μm) and usually 1–2 celled.

*Type*: **Pakistan**: *Khyber Pakhtoon Khaw*: Abbottabad, Shimla, 34°10′27.62“ N, 73°12’18.13” E, alt. 1297 m, under *Pinus roxburghii*, 14 Sept. 2012, *M. Saba & A.N. Khalid MSM#0072* (FH00304589 – holotype).

*Description*: *Basidiomata* small to medium-sized, relatively thin-fleshed. *Pileus* 13–73 mm diam, first convex, then plano-convex to applanate, sometimes slightly centrally depressed; margin first deflexed, when mature usually inflexed, entire, hardly striated; pileus cuticle matt when dry, sometimes viscid and shiny near the centre; colour moderate red, strong red, deep red, vivid red or vivid reddish orange, rarely with light orange-yellow spots near the centre. *Lamellae* adnexed or adnate-emarginate, brittle, without lamellulae; when young white to pale yellow, when mature yellow; edge even, concolourus. *Stipe* 16–35 × 7–12 mm, central, clavate or cylindrical, longitudinally rugulose, not pruinose, white, occasionally with pink flush, with yellowish brown or rusty spots, solid. *Context* compact, white, slowly turning yellow-brownish when wounded or old. *Smell* indistinct. *Taste* mild to slightly acrid. *Spore print* yellow.

*Spores* (8.3–)9.1–9.9–10.6(− 11.4) × (6.9–)7.6–8.3–9(− 9.7) μm, broadly ellipsoid, Q = (1.14–)1.17–1.19–1.21(− 1.23), ornamentation of large, moderately distant [3–6(− 7) in a 3 μm diam circle] amyloid warts, (0.8–)0.9–1.2(− 1.5) μm high; occasionally fused in pairs or short chains [0–2(− 3) fusions in the circle], line connections very rare and short [0–1(− 2) in the circle], warts mainly isolated; suprahilar plage large, amyloid. *Basidia* (35–)36.5–39–41(− 42) × (12–)12.5–13.9–15(− 15.5) μm, 4-spored, broadly clavate, pedicellate; basidiola first cylindrical, then clavate, *ca* 4–12 μm wide. *Subhymenium* pseudoparenchymatic. *Lamellar trama* mainly composed of large sphaerocytes. *Pleurocystidia* moderately numerous, *ca* 950–1150/mm^2^, (44–)52–60.6–69(− 89) × (9.5–)11–12.3–13.5(− 14.5) μm, fusiform or clavate, pedicellate, apically usually subacute, often with short 1–2(− 4) μm long appendage, thin-walled, contents heteromorphous-banded, turning dark brown-black in sulfovanillin. *Cheilocystidia* abundant, (32–)44.5–54.1–63.5(− 82) × (7–)8–9.5–11(− 13) μm, clavate, pedicellate, apically mainly obtuse-rounded, contents banded or granulose. *Marginal cells* smaller, but usually similar to basidiola on gill sides, subcylindrical or clavate, (8–)10–16.6–23(− 35) × (3.5–)4–5.4–6.5(− 9) μm, apically obtuse. *Pileipellis* orthochromatic in Cresyl blue, not sharply delimited from the underlying sphaerocytes of the context, strongly gelatinized in all parts, 165–225 μm deep, vaguely divided in 60–75 μm deep suprapellis of erect, densely arranged hyphae, gradually transitioning into a 100–120 μm deep subpellis of intricate, relatively loose, irregularly oriented, but near the trama horizontally oriented and dense, 1.5–3 μm wide hyphae. Acid-resistant incrustations absent, but contents of pileocystidia stain red after carbol fuchsin treatment. Hyphal terminations near the pileus margin slender, often slightly moniliform or flexuous, thin-walled, with terminal cells (18–)25.5–32.9–40.5(− 50) × (2–)2.5–2.9–3.5(− 4) μm, cylindrical or subulate, apically frequently constricted or attenuated, less frequently obtuse; subterminal cells mainly branched, often flexuous and intricate. Hyphal terminations near the pileus centre similar, with terminal cells (26–)29.5–38–46.5(− 55) × (2–)2.5–2.8–3(− 3.5) μm, apically usually constricted or attenuated. *Pileocystidia* near the pileus margin numerous, 1–1.6–2(− 3) celled, thin-walled, narrow, terminal cells (16–)22–45–68(− 91) × (3–)3.5–4.6–5.5(− 6) μm, cylindrical, apically obtuse, contents abundant heteromorphous, banded, turning black-brown in sulfovanillin; near the pileus centre with often shorter terminal cells, (12–)14–29.1–44(− 70) × (3.5–)4–4.8–5.5(− 6) μm; subterminal cells usually equally wide but often distinctly longer. Cystidioid hyphae present only in the upper part of the subpellis, absent in the pileus trama. *Clamp connections* absent in all parts.

*Additional material examined:* **Pakistan**: *Khyber Pakhtoon Khaw*: Abbottabad, Shimla, 34°10′27.62“ N 73°12’18.13” E, alt. 1297 m, under *Pinus roxburghii*, 14 Sept. 2012, *M. Saba & A.N. Khalid MSM#0073* (LAH310071); ibid., 4 Aug. 2014, *M. Saba & A.N. Khalid MSM#00151* (LAH310099); Mansehra Batrasi, 34°23′42.94“ N 73°18’53.03” E, alt. 1113 m, under *Pinus roxburghii*, 3 Aug. 2014, *M. Saba & A.N. Khalid MSM#0078* (FH00304558).

## DISCUSSION

This study confirms that *Russula dryadicola* is a well-defined species in accordance with Fellner & Landa (1993), contrary to some previous studies that treat it as an infraspecific taxon of *R. maculata* (Singer 1926, Knudsen & Borgen 1992b). The species was first reported as *R. maculata* subsp. *alpina* from the Alps (Singer 1925, 1926) and considered an alpine-arctic element distributed in Greenland, Scandinavia, mountains of Central Europe and the Ural Mts (Kühner 1975, Knudsen & Borgen 1992b). The related European species *Russula globispora* differs in having larger spores and, based on this, collections from mixed boreal forests (mainly with *Picea* and *Betula*) in southern Finland were reported by J. Ruotsalainen and J. Vauras under the name *R. globispora* (Sarnari 1998). We have studied seven collections sent by J. Vauras from similar boreal habitats in Finland, and they all morphologically correspond to *R. dryadicola.* In our phylogenetic tree, boreal collections are represented by TURA 152390 and TU 101835, and they are clustered with other alpine and arctic collections of *R. dryadicola*. This species is an example of alpine-arctic habitats being incorrectly applied for species delimitation and identification. Similarly, *R. nuoljae*, previously described and recorded from alpine and arctic habitats, was recently reported also from boreal forests, and *R. subrubens*, previously reported only from lowlands of the temperate belt vegetated by willows, was synonymized with the alpine-arctic *R. chamitae* (Adamčík et al. 2016a). As new morphological characters of *R. dryadicola*, differentiating it from *R. globispora* (the latter species is re-described in Adamčík & Jančovičová 2013), we introduce here shorter (on average to 33 μm long) and often fusiform terminal cells of hyphae in the pileipellis near the pileus margin, narrower pileocystidia (on average to 6.5 wide; Fig. 3) and narrower pleurocystidia (on average to 12.5 μm wide; Fig. 4). Alaskan samples from boreal forests have nearly identical ITS sequences and any decision about their taxonomic status requires support of more DNA loci or other molecular support. There are also two ITS sequences from environmental samples associated with the alpine sedge *Kobresia myosuroides*; these sequences are of unspecified origin and have apparently been sampled for the Niwot Ridge project (http://niwot.colorado.edu) dealing with research of permanent plots in the Colorado alpine ecosystem in the Rocky Mountains (USA). Nevertheless, this affinity of North American and European collections suggests a wide distribution of *R. dryadicola* in Eurasian taiga and tundra habitats, as demonstrated by Geml et al. (2012). North American ITS sequences from environmental DNA samples, placed in this study in the *R. globispora* lineage, are the first reports of the group from America, but not a single basidiome has been collected in that region to date.

The BLAST result of the ex-holotype ITS sequence of *R. tengii* shows 99% identity (under 100% query coverage) with the sequence of the *R. dryadicola* collection TURA 151632. This is an example of high similarity in the ITS region within the *R. globispora* clade. However, all three species recognized in our multi-locus phylogenetic analysis (Fig. 1) received good support (BS ≥ 65, PP ≥ 0.98). This is analogous to the high ITS sequence similarity within the *R. clavipes* species complex (Adamčík et al. 2016a). *Russula tengii* is represented only by collections from mixed subalpine forests of the southwest Himalayan Mountains in China. This type of habitat falls within the known ecological amplitude of *R. dryadicola*. The existence of allopatric boreal-arctic species within the *R. globispora* clade suggests that glaciation events and geographic and climate disjunctions were the evolutionary drivers of speciation in the group on the Eurasian scale. It is possible that the common ancestor of *R. dryadicola* and *R. tengii* migrated via available corridors for movement to newly emerging environments during climatic changes in the Quaternary (Donoghue 2008), but we do not know the original area of this ancestor species.

The collections of *R. abbottabadensis* under study originate from subtropical coniferous forests of *Pinus roxburghii* in northeast Pakistan at the foothills of the Himalayas. Coniferous trees seem to be obvious hosts of this species, and they possibly also host *R. dryadicola* and *R. tengii*, which grow in mixed boreal forests. The long branch of this species and lack of support for *R. globispora* suggest missing data on possibly unrepresented species of the *R. globispora* lineage from other environments and regions of Eurasia. It is also possible that gene flow and species migrations between Southeast Asia and Europe were multidirectional and that *R. abbottabadensis* may represent another lineage of so far unknown origin. Both Asian species described here represent, to our knowledge, the first reports of the *R. globispora* lineage from Southeast Asia and we hope that this study will boost more studies from different parts of the continent to answer questions on origin and speciation pattern of this *Russula* group.

Looney et al. (2016) suggested that host switching plays major role for species diversification of the genus *Russula* especially at the rank of clades. However, the speciation mechanism of *Russulaceae* and even ECM fungi in general is not well understood especially within closely related species lineages. This study suggests that host switch did not contribute to speciation of studied species of *R. globispora* lineage and more probable drivers in the evolution of *R. tengii* and *R. abbottabadensis* were climate disjunctions and adaptations.

## CONCLUSIONS

We recognised three species of R. globispora lineage that either grow in mixed forests in cold climate (boreal, montane, arctic or alpine) or are associated with conifers in mountains. *Russula dryadicola* is widely distributed in alpine, arctic and boreal areas of Eurasia and Northern America, although North American collections show little genetic divergence. This species proved the hypothesis of widely distributed ECM species in hemiboreal areas (Geml et al. 2012). Collections from south Himalayas represent two species distinct by its morphology, genetics, climate and ecological preferences; they are described here as *R. abbottabadensis* and *R. tengii*. The results suggest that climatic disjunction and isolation by distance were drivers of speciation within the lineage. The host range and distribution limits of the studied species are not sufficiently known yet and the research in this area may contribute to understanding of evolutionary processes of ECM fungi.

## Additional file

Additional file 1: **Table S1.** List of samples with collection details and GenBank numbers of corresponding DNA sequences. Sequences starting with MG are published first in this study. (DOCX 33 kb)

## References

[CR1] ADAMČÍK SLAVOMÍR, CABOŇ MIROSLAV, EBERHARDT URSULA, SABA MALKA, HAMPE FELIX, SLOVÁK MAREK, KLEINE JESKO, MARXMÜLLER HELGA, JANČOVIČOVÁ SOŇA, PFISTER DONALD H., KHALID ABDUL N., KOLAŘÍK MIROSLAV, MARHOLD KAROL, VERBEKEN ANNEMIEKE (2016). A molecular analysis reveals hidden species diversity within the current concept of Russula maculata (Russulaceae, Basidiomycota). Phytotaxa.

[CR2] Adamčík S, Jančovičová S (2013). Type studies in *Russula* subsection *Maculatinae* and affiliated taxa: four species as interpreted by Henri Romagnesi. Sydowia.

[CR3] Adamčík S, Marhold K (2000). Taxonomy of the *Russula xerampelina* group. I. Morphometric study of the *Russula xerampelina* group in Slovakia. Mycotaxon.

[CR4] Adamčík S, Slovák M, Eberhardt U, Ronikier A, Jairus T (2016). Molecular inference, multivariate morphometrics and ecological assessment are applied in concert to delimit species in the *Russula clavipes* complex. Mycologia.

[CR5] Bahram M, Põlme S, Koljalg U, Zarre S, Tedersoo L (2012). Regional and local patterns of ectomycorrhizal fungal diversity and community structure along an altitudinal gradient in the Hyrcanian forests of northern Iran. New Phytologist.

[CR6] Bazzicalupo AL, Buyck B, Saar I, Vauras J, Carmean D, Berbee ML (2017). Troubles with mycorrhizal mushroom identification where morphological differentiation lags behind barcode sequence divergence. Taxon.

[CR7] Biscoletto A, Ostellari C (2005) *Russula dryadicola* (Singer) Fellner & Landa, primo ritrovamento in Italia. Rivista di Mycologia 4:301–307

[CR8] Buyck B (1989) Valeur taxonomique du bleu de crésyl pour le genre *Russula*. Bulletin Trimestriel de la Societé Mycologique de France 105:1–6

[CR9] Buyck B (1991). The study of microscopic features in *Russula* 2. Sterile cells of the hymenium. Russulales News.

[CR10] Caboň M, Eberhardt U, Looney BP, Hampe F, Kolařík M (2017). New insights in *Russula* subsect. *Rubrinae*: phylogeny and the quest for synapomorphic characters. Mycological Progress.

[CR11] Cao Y, Zhang Y, Yu Z, Mi F, Liu C (2013). Structure, gene flow, and recombination among geographic populations of a *Russula virescens* ally from southwestern China. PLoS One.

[CR12] Das K, Dowie NJ, Li GJ, Miller SL (2014). Two new species of *Russula* (*Russulales*) from India. Mycosphere.

[CR13] Das K, Ghosh A, Bhatt RP, Chakraborty D, Hofstetter V, Buyck B (2017a) Fungal biodiversity profiles 41–50. Cryptogamie, Mycologie 38:527–547

[CR14] Das K, Ghosh A, Chakraborty DF, Li J, Qiu L et al (2017b) Fungal biodiversity profiles 31–40. Cryptogamie, Mycologie. Mycologie 38:353–406

[CR15] Donoghue MJ (2008). A phylogenetic perspective on the distribution of plant diversity. Proceedings of the National Academy of Sciences, USA.

[CR16] Eberhardt U, Kress WJ, Erickson DL (2012). Methods for DNA barcoding of fungi. DNA barcodes methods and protocols.

[CR17] Fellner R, Landa J (1993). Some species of *Cortinariaceae* and *Russulaceae* in the alpine belt of the Belaer Tatras. Bibliotheca Mycologica.

[CR18] Feng G, Mi X, Eiserhardt WL, Jin G, Sang W (2015). Assembly of forest communities across East Asia insights from phylogenetic community structure and species pool scaling. Scientific Reports.

[CR19] Gardes M, Bruns TD (1993). ITS primers with enhanced specificity for basidiomycetes-application to the identification of mycorrhizae and rusts. Molecular Ecology.

[CR20] Geml J, Timling I, Robinson CH, Lennon N, Nusbaum HC (2012). An arctic community of symbiotic fungi assembled by long-distance dispersers: phylogenetic diversity of ectomycorrhizal basidiomycetes in Svalbard based on soil and sporocarp DNA. Journal of Biogeography.

[CR21] Gouy M, Guindon S, Gascuel O (2010). SeaView version 4: a multiplatform graphical user interface for sequence alignment and phylogenetic tree building. Molecular Biology and Evolution.

[CR22] Hongo T (1960). The *Agaricales* of Japan I-3. *Russulaceae*. Acta Phytotaxonomica Geobotanica.

[CR23] Joshi S, Bhatt RP, Stephenson SL (2012). The current status of the family *Russulaceae* in the Uttarakhand Himalaya, India. Mycosphere.

[CR24] Katoh K, Standley DM (2013). MAFFT multiple sequence alignment software, version 7: improvements in performance and usability. Molecular Biology and Evolution.

[CR25] Kearse M, Moir R, Wilson A, Stones-Havas S, Cheung M (2012). Geneious basic: an integrated and extendable desktop software platform for the organization and analysis of sequence data. Bioinformatics.

[CR26] Knudsen H, Borgen T (1992). New and rare taxa of *Russula* from Greenland. Persoonia.

[CR27] Knudsen H, Borgen T (1992b) *Russulaceae* in Greenland. In: *Arctic and Alpine Mycology* (Laursen GA, Ammirati JF, eds) **1**: 216–244. Seattle: University of Washington Press

[CR28] Kühner R (1975) *Agaricales* de la zone Alpine. Genre *Russula* Pers, ex S. F. Gray. Bulletin Trimestriel de la Societé Mycologique de France 91:313–390

[CR29] Lanfear R, Calcott B, Ho SY, Guindon S, Lanfear R (2012). PartitionFinder: combined selection of partitioning schemes and substitution models for phylogenetic analyses. Molecular Biology and Evolution.

[CR30] Li GJ, Hyde KD, Zhao RL, Hongsanan S, Abdel-Aziz FA (2016). Fungal diversity notes 253–366: taxonomic and phylogenetic contributions to fungal taxa. Fungal Diversity.

[CR31] Li GJ, Li SF, Liu XZ, Wen HA (2012). *Russula jilinensis* sp. nov. (*Russulaceae*) from Northeast China. Mycotaxon.

[CR32] Looney BP, Meidl P, Piatek MJ, Miettinen O, Martin FM (2018). *Russulaceae*: a new genomic dataset to study ecosystem function and evolutionary diversification of ectomycorrhizal fungi with their tree associates. New Phytologist.

[CR33] Looney BP, Ryberg M, Hampe F, Sánchez-García M, Matheny PB (2016). Into and out of the tropics: global diversification patterns in a hyperdiverse clade of ectomycorrhizal fungi. Molecular Ecology.

[CR34] Marstad P (2004). Russula in the Nordic countries. Tønsberg: P Marstad.

[CR35] Matheny PB (2005). Improving phylogenetic inference of mushrooms with RPB1 and RPB2 nucleotide sequences (*Inocybe*; *Agaricales*). Molecular Phylogenetics and Evolution.

[CR36] Miehe G, Miehe S, Böhner J, Bäumler R, Ghimire SK, Miehe G, Pendry CA, Chaudhary R (2015). Vegetation ecology. An Introduction to the natural history, ecology and human environment of the Himalayas Nepal.

[CR37] Põlme S, Bahram M, Yamanaka T, Nara K, Dai YC (2013). Biogeography of ectomycorrhizal fungi associated with alders (*Alnus* spp.) in relation to biotic and abiotic variables at the global scale. New Phytologist.

[CR38] Rambaut A, Suchard MA, Xie D, Drummond AJ (2013) *Tracer*. Version 1.6. http://beast.bio.ed.ac.uk/software/tracer/

[CR39] Romagnesi H (1967). Les Russules d’Europe et d'Afrique du Nord.

[CR40] Ronquist F, Teslenko M, van der Mark P, Avres DL, Darling A (2012). MrBayes 3.2: efficient Bayesian phylogenetic inference and model choice, across a large model space. Systematic Biology.

[CR41] Ruotsalainen J, Vauras J (1994). Novelties in *Russula* : *R. olivobrunnea*, *R. intermedia* and *R. groenlandica*. Karstenia.

[CR42] Ryberg M (2015). Molecular operational taxonomic units as approximations of species in the light of evolutionary models and empirical data from *Fungi*. Molecular Ecology.

[CR43] Sarnari M (1998). Monographia Illustrata del Genere Russula in Europa.

[CR44] Silvestro Daniele, Michalak Ingo (2011). raxmlGUI: a graphical front-end for RAxML. Organisms Diversity & Evolution.

[CR45] Singer R (1925). Zur *Russula*-Forschung. Zeitschrift für Pilzkunde.

[CR46] Singer R (1926). Monographie der Gattung Russula. Hedwigia.

[CR47] Singer R (1938) Contribution a l’étude des Russules (1). 3. Quelques Russules américaines et asiatiques. Bulletin de la Société Mycologique de France Fr 54:132–177

[CR48] Stamatakis A (2008) The RAxML 7.0.4 manual. https://web.natur.cuni.cz/~vlada/moltax/RAxML-Manual.7.0.4.pdf

[CR49] Tian H, Liu T, Lian J, Li G, Ba T (2014). Identification and classification of four *Russula* species from Inner Mongolia based on morphology and ITS sequencing. Acta Edulis Fungi.

[CR50] White TJ, Bruns T, Lee S, Taylor J, Innis MA, Gelfand DH, Sninsky JJ, White TJ (1990). Amplification and direct sequencing of fungal ribosomal RNA genes for phylogenetics. PCR Protocols: a guide to methods and applications.

