# Supplementary material for: Phylogenetic study documents different speciation mechanisms within the Russula globispora lineage in boreal and arctic environments of the Northern Hemisphere
Source: IMA Fungus. 2019 Jun 7;10:5. doi: 10.1186/s43008-019-0003-9 (PMC7325667; doi:10.1186/s43008-019-0003-9)
Supplement: Supplementary file 1 — Table S1. List of samples with collection details and GenBank numbers of corresponding DNA sequences. Sequences starting with MG are published first in this study. (DOCX 33 kb) [file 43008_2019_3_MOESM1_ESM.docx]

Additional file 1: Table S1. List of samples with collection details and GenBank numbers of corresponding DNA sequences. Sequences starting with MG are published first in this study.

| **Species (orig. identification)** | **Herbarium acronym (collection number)** | **Country** | **Collecting area** | **Host** | **Collector** | **Date** | **ITS** | **mtSSU** | **RPB2** |
| --- | --- | --- | --- | --- | --- | --- | --- | --- | --- |
| *R. abbottabadensis* | FH00304558 | Pakistan | Khyber Pakhtoon Khaw: Mansehra Batrasi, 34°23'42.94" N, 73°18'53.03" E, alt. 1113 m | under *Pinus roxburghii* | M. Saba, A.N. Khalid | 3 Aug 2014 | MG386705 | MG386721 | MG386738 |
| *R. abbottabadensis* | FH00304589 | Pakistan | Khyber Pakhtoon Khaw: Abbottabad, Shimla, 34°10'27.62" N, 73°12'18.13" E, alt. 1297 m | under *Pinus roxburghii* | M. Saba, A.N. Khalid | 14 Sep 2012 | MG386704 | MG386720 | - |
| *R. abbottabadensis* | LAH310071 | Pakistan | Khyber Pakhtoon Khaw: Abbottabad, Shimla, 34°10'27.62" N, 73°12'18.13" E, alt. 1297 m | under *Pinus roxburghii* | M. Saba, A.N. Khalid | 14 Sep 2012 | MG386703 | MG386719 | MG386737 |
| *R. abbottabadensis* | LAH310099 | Pakistan | Khyber Pakhtoon Khaw: Abbottabad, Shimla, 34°10'27.62" N, 73°12'18.13" E, alt. 1297 m | under *Pinus roxburghii* | M. Saba, A.N. Khalid | 4 Aug 2014 | MG386702 | MG386718 | MG386736 |
| *R. adulterina* | SAV F-4451 | Poland | W Carpathians: Droga pod Reglami | *Fagus*, *Picea* | S. Adamčík | 25 Aug 2014 | MG386710 | MG386730 | MG386750 |
| *R. cuprea* | GENT (2010 BT168) | Germany | Thuringia: forest near Nohra | *Fagus*, on limestone | F. Hampe | 20 Aug 2010 | MG386711 | MG386731 | MG386751 |
| *R. dryadicola* | (TS1473) | Estonia | Ida-Viru: Illuka | - | - | - | UDB014592 | - | - |
| *R. dryadicola* | TU 101835 | Estonia | Lääne-Viru: S side of Rakke, at the road to Laululava | rich forest with *Betula, Picea abies, Populus tremula* and *Corylus avellana* | J. Vauras | 7 Sep 2011 | UDB016122 | - | - |
| *R. dryadicola* | TURA 151632 | Finland | Lapland: Kilpisjärvi, Saana, near the biological station | birch forest | J. Ruotsalainen | 16 Aug 1990 | KU928141 | KY471623 / MG386728 | KY616724 / MG386745 |
| *R. dryadicola* | TURA 152390 | Finland | Etelä-Häme: Raikonkulma, Raikko, E of Kivijärvi lake, Kalkkimäki | herb rich forest with *Picea, Pinus, Betula, Salix caprea, Populus tremula* | J. Vauras | 22 Aug 2003 | MG386712 | MG386729 | MG386746 |
| *R. dryadicola* | LIP (PAM00082907) | France | Savoie: Bourg-Saint-Maurice, Arc 2000, vers col des Frettes | alpine pasture with *Dryas octopetala,* on dolomite | P.-A. Moreau | 29 Aug 2000 | MG386713 | MG386723 | MG386740 |
| *R. dryadicola* | LIP (PAM95082603) | France | Savoie: Bourg-Saint-Maurice, Arc 2000, vers col des Frettes | alpine pasture with *Dryas octopetala,* on dolomite | P.-A. Moreau | 26 Aug 1995 | MG386715 | MG386724 | MG386741 |
| *R. dryadicola* | LIP (PAM98082511) | France | Savoie: Bourg-Saint-Maurice, Arc 2000, vers col des Frettes | alpine pasture with *Dryas octopetala,* on dolomite | P.-A. Moreau | 25 Aug 1998 | MG386716 | MG386725 | MG386742 |
| *R. dryadicola* | MCVE 16243 | France | - | - | L. Levorato | 24 Aug 2001 | JF908710 | - | - |
| *R. dryadicola* | IB 2002/0432 | Italy | Südtirol: Sexten, Fischleintal, oberhalb Zsigmondyhütte, ca. 2400 m | - | U. Peintner, I. Göschl | 5 Aug 2006 | KU928145 | MG386722 | MG386739 |
| *R. dryadicola* | (F2D35_11) | Norway | Finse: Sandalsnuten | *Dryas octopetala* (root sample) | - | summer 2006 - 2007 | HQ445043 | - | - |
| *R. dryadicola* | (F2D35_12) | Norway | Finse: Sandalsnuten | *Dryas octopetala* (root sample) | - | summer 2006 - 2007 | HQ445044 | - | - |
| *R. dryadicola* | (F2D35_17) | Norway | Finse: Sandalsnuten | *Dryas octopetala* (root sample) | - | summer 2006 - 2007 | HQ445046 | - | - |
| *R. dryadicola* | (F2D35_9) | Norway | Finse: Sandalsnuten | *Dryas octopetala* (root sample) | - | summer 2006 - 2007 | HQ445041 | - | - |
| *R. dryadicola* | UPS (AT2004140) | Sweden | Uppsala: Nåsten | probably with birch | A. Taylor | 23 Sep 2004 | KU928146 |  | MG386747 |
| *R. globispora* | GENT (2007 BT111) | Germany | Thuringia: Gügleben, Werningslebener Wald | - | F. Hampe | 8 Sep 2007 | KU928144 | KY471564 | KY616671 |
| *R. globispora* | GENT (2007 BT121) | Germany | Thuringia: Gügleben, Werningslebener Wald | - | J. Girwert | 4 Sep 2007 | KU886594 | MG386726 | MG386743 |
| *R. globispora* | GENT (2007 BT98) | Germany | Thuringia: Bechstedter Holz near Bechstedt-Wagd | - | F. Hampe | 4 Aug 2007 | MG386717 | MG386727 | MG386744 |
| *R. juniperina* | SAV F-4998 | Italy | Tuscany: Marsiliana Riserva Statale | *Quercus suber*, *Q. ilex* | L. Michelin | 9 Nov 2016 | MG386714 | - | MG386749 |
| *R. maculata* | GENT (2011 BT005) | Germany | Thuringia: Palace Garden Gotha | *Tilia*, *Carpinus*, *Quercus* | F. Hampe | 5 Jul 2011 | KU928152 | - | MG386748 |
| *Russula sp.* | (clone 5) | Canada | Alberta: North-East Alberta, near Conklin | *Populus tremuloides* | Stefan Hupperts | 2014 | KX379149 | - | - |
| *Russula sp.* | (K68 clone 7) | USA | - | *Kobresia myosuroides* | - | - | AF495465 | - | - |
| *Russula sp.* | (K68 clone 2) | USA | - | *Kobresia myosuroides* | - | - | AF495464 | - | - |
| *Russula sp.* | (UP1_3165_F18) | USA | Interior Alaska: Bonanza Creek Long Term, Ecological Research Site, Parks Hwy | early-successional upland mixed forest | - | - | EU711796 | - | - |
| *Russula sp.* | (UP1_3166_O6) | USA | Interior Alaska: Bonanza Creek Long Term, Ecological Research Site, Parks Hwy | early-successional upland mixed forest | - | - | EU711801 | - | - |
| *Russula sp.* | (UP1_3166_B10) | USA | Interior Alaska: Bonanza Creek Long Term, Ecological Research Site, Parks Hwy | early-successional upland mixed forest | - | - | EU711800 | - | - |
| *Russula sp.* | (UP3_3195_M19) | USA | Interior Alaska: Bonanza Creek Long Term, Ecological Research Site, Parks Hwy | late-successional upland mixed forest | - | - | EU711847 | - | - |
| *Russula sp.* | (F_UP1_3157_O10) | USA | Interior Alaska: Bonanza Creek Long Term, Ecological Research Site, Parks Hwy | early-successional upland mixed forest | - | - | EU711757 | - | - |
| *Russula sp.* | (UP4_3209I20) | USA | Interior Alaska: Bonanza Creek Long Term, Ecological Research Site, Parks Hwy | early-successional upland mixed forest | - | - | EU711876 | - | - |
| *Russula sp.* | (UP4_3214F3) | USA | Interior Alaska: Bonanza Creek Long Term, Ecological Research Site, Parks Hwy | early-successional upland mixed forest | - | - | EU711878 | - | - |
| *Russula sp.* | (UP1_3165_I23) | USA | Interior Alaska: Bonanza Creek Long Term, Ecological Research Site, Parks Hwy | early-successional upland mixed forest | - | - | EU711798 | - | - |
| *Russula sp.* | (UP4_3220F23) | USA | Interior Alaska: Bonanza Creek Long Term, Ecological Research Site, Parks Hwy | early-successional upland mixed forest | - | - | EU711885 | - | - |
| *R. tengii* | (3367) | China | - | - | - | - | KP226186 | - | - |
| *R. tengii* | HMAS244255 | China | Xizang Autonomous Region, Maizhokunggar County, Riduo Township, 29°38′ N, 92°26′ E | *Abies*, *Betula* | W.L. Lu | 6 Aug 2012 | MG386709 | MG386735 | MG386755 |
| *R. tengii* | HMAS251829 | China | Xizang Autonomous Region, Maizhokunggar County, Gesang village, 29°48′ N, 92°39′ E | *Abies*, *Betula* | G.J. Li, D. Zhao, W. Li | 16 Aug 2012 | MG386706 | MG386732 | MG386752 |
| *R. tengii* | HMAS262728 | China | Xizang Autonomous Region, Riwoqe County, 31°12′ N, 96°36′ E, alt. 3741 m |  | T.Z. Wei | 24 Jul 2010 | MG386708 | MG386734 | MG386754 |
| *R. tengii* | HMAS264837 | China | Xizang Autonomous Region, Maizhokunggar County, Gesang village, 29°48′ N, 92°39′ E | *Abies*, *Betula* | G.J. Li, D. Zhao, S. Qi | 16 Aug 2012 | MG386707 | MG386733 | MG386753 |
